# Effects of Dietary Maltol on Innate Immunity, Gut Health, and Growth Performance of Broiler Chickens Challenged With *Eimeria maxima*

**DOI:** 10.3389/fvets.2021.667425

**Published:** 2021-05-20

**Authors:** Inkyung Park, Doyun Goo, Hyoyoun Nam, Samiru S. Wickramasuriya, Kichoon Lee, Noah P. Zimmerman, Alexandra H. Smith, Thomas G. Rehberger, Hyun S. Lillehoj

**Affiliations:** ^1^Animal Bioscience and Biotechnology Laboratory, Beltsville Agricultural Research Center, Agricultural Research Service, United States Department of Agriculture, Beltsville, MD, United States; ^2^Department of Animal Sciences, The Ohio State University, Columbus, OH, United States; ^3^Arm & Hammer Animal and Food Production, Waukesha, WI, United States

**Keywords:** broiler chickens, *Eimeria maxima*, growth performance, gut health, maltol, metabolites, postbiotics

## Abstract

Two studies were conducted to evaluate the effects of maltol as a postbiotic on innate immunity, gut health, and enteric infection. In the first study, an *in vitro* culture system was used to evaluate the effects of maltol on the innate immune response of chicken macrophage cells (CMC), gut integrity of chicken intestinal epithelial cells (IEC), anti-parasitic activity against *Eimeria maxima*, and differentiation of quail muscle cells (QMC) and primary chicken embryonic muscle cells (PMC). All cells seeded in the 24-well plates were treated with maltol at concentrations of 0.1, 1.0, and 10.0 μg. CMC and IEC were stimulated by lipopolysaccharide to induce an innate immune response, and QMC and PMC were treated with 0.5 and 2% fetal bovine serum, respectively. After 18 h of incubation, pro-inflammatory cytokines, tight junction proteins (TJPs), and muscle cell growth markers were measured. In the second study, the dietary effect of maltol was evaluated on disease parameters in broiler chickens infected with *E. maxima*. Eighty male 1-day-old broiler chickens were allocated into the following four treatment groups: (1) Control group without infection, (2) Basal diet with *E. maxima*, (3) High maltol (HI; 10.0 mg /kg feed) with *E. maxima*, and (4) Low maltol (LO; 1.0 mg/kg feed) with *E. maxima*. Body weights (BW) were measured on days 0, 7, 14, 20, and 22. All chickens except the CON group were orally infected with 10^4^ *E. maxima* per chicken on day 14. Jejunum samples were collected for gut lesion scoring, and the gene expression of cytokines and TJPs. Data was analyzed using PROC MIXED in SAS. *In vitro*, maltol not only increased TJPs in IEC and cytokines in the LPS-stimulated CMC but also showed direct cytotoxicity against sporozoites of *E. maxima*. *In vivo*, the HI group improved the BW, reduced the gut lesion scores and fecal oocyst shedding, and decreased jejunal TNFSF15 and IL-1β expression in *E. maxima-*infected chickens. In conclusion, these results demonstrate the beneficial effects of dietary maltol in the enhancement of growth performance, gut health, and coccidiosis resistance and the applicability of maltol as a postbiotic for the replacement of antibiotic growth promoters in commercial poultry production.

## Introduction

*Eimeria* spp. are the etiologic agents of avian coccidiosis, an intestinal disease responsible for an economic loss of more than $3 billion per year (1, 2). Increasing implementation of antibiotic-free poultry production system in the U.S. is making the control of some enteric pathogens such as coccidiosis-causing *Eimeria* species and necrotic enteritis-inducing *Clostridium perfringens* strains challenging (3). Because coccidiosis is a primary risk factor for necrotic enteritis, it is desirable if alternatives to antibiotics can reduce *Eimeria* as well as *Clostridium perfringens* (4). Development of antibiotic alternatives is a priority for the animal agriculture industry to maintain the growth performance and health of food-producing animals without losing productivity in the post-antibiotic era (5). Although the mode of action of antibiotic growth promoters remains debatable, dietary antibiotic growth promoters undoubtedly influence the gut microbiota, host immune status, and other host physiological responses (2–4). Therefore, developing antibiotic alternatives that can manipulate gut microbiota to promote host growth and health is a logical goal for the animal industry (6, 7). Considering this goal, metagenomics has been conducted in several studies to analyze the entire population of gut microbiota based on various research areas such as nutrition, physiology, and immunology (8, 9). Although amplicon sequencing using metagenomics enables comprehensive characterization of the taxonomic composition of the gut microbiome, it is impossible to obtain direct evidence of the microbial functions related to the gut microbial community (10, 11). Hence, the critical next step for gut microbiome studies is a transition from gene/genome-centric analysis to mechanism-centric techniques by integrating omics data with experimental results (12, 13). Thus, mainstream studies on the gut microbiota include adoption of a combination of analytical approaches, such as meta-transcriptomics, meta-proteomics, and metabolomics, together with established metagenomics (14–17). With the development of the “omics” technology related to the investigation of gut health, the term “postbiotics” was coined and defined as a novel class of feed additives that are generally produced by beneficial gut microbes and which exert a positive influence on the host health (18). In our previous study (19), dietary feeding of the *Bacillus subtilis* 1781 strain induced the alteration of chicken gut metabolites which was associated with the growth- and immune-promoting effects of dietary *B. subtilis* 1781 strain in chickens. Among the highly altered gut metabolites, maltol was one of the significantly increased metabolites that we selected for further studies since maltol mediates various physiological functions associated with anti-oxidant (20) and anti-carcinogen (21) activities and reduces inflammatory responses (22).

We hypothesized that maltol could be a good candidate as a growth-promoting postbiotic because its expression was highly upregulated in the gut of chickens fed with a diet supplemented with the *B. subtilis* strain 1781, which influenced the intestinal immune response, gut barrier integrity, and growth-promotion in fast-growing chickens. Therefore, the objectives of the present study were: (1) the *in vitro* evaluation of effects of maltol on the host innate immune response using chicken macrophage cells (CMC), on the barrier integrity on chicken intestinal epithelial cells (IEC), on anti-coccidial ability against *Eimeria maxima*, and on myogenic differentiation of quail muscle cells (QMC) and primary chicken embryonic muscle cells (PMC); and (2) the *in vivo* characterization of the dietary maltol on growth performance, intestinal immunity, and epithelial integrity in young broiler chickens challenged with *E. maxima*. These studies will provide information on the mechanisms through which the alteration of gut metabolites generated by dietary direct-fed microbes exerts beneficial physiological changes on the host.

## Materials and Methods

### Experiment 1: *In vitro* Study

#### Culture of Chicken Intestinal Epithelial Cells (IEC) and Chicken Macrophage Cells (CMC)

IEC (2 × 10^5^/mL, 8E1; MicroMol GmbH, Karlsruhe, Germany) and CMC (2 × 10^5^/mL, HD11) (23) were seeded in 24-well plates and grown in the Dulbecco's modified Eagle medium (DMEM)/F-12 (Hyclone, Logan, UT) supplemented with 1% penicillin/streptomycin (Gibco, Grand Island, NY, USA) and 10% heat-inactivated fetal bovine serum (FBS, Hyclone), and incubated at 41°C in a humidified atmosphere with 5% CO_2_ for 24 h for cell adhesion. After 24 h, lipopolysaccharide (LPS, Sigma-Aldrich, St. Louis, MO) at a concentration of 1.0 μg/mL and maltol (Sigma-Aldrich) at concentrations of 0.0, 0.1, 1.0, and 10.0 μg/mL were added to each well in the 24-well plates. After 18 h, all cells were harvested in lysis buffer (QIAGEN, Hilden, Germany) with 2-mercaptoethanol (Sigma-Aldrich). RNA was extracted using QIAcube (QIAGEN) for performing quantitative real-time polymerase chain reaction (qRT-PCR) analysis. All experiments were replicated more than three times independently.

#### Anticoccidial Assay Against *E. maxima*

Sporozoites of *E. maxima* were obtained by excystation of sporulated oocysts as described (24). Briefly, fresh sporulated oocysts were disrupted with 0.5-mm glass beads for 10 s using the Mini-Beadbeater (BioSpec Products, Bartlesville, OK). The released sporocysts were washed in chilled Hanks' balanced salt solution (Hyclone) and treated with 0.25% trypsin and 0.014 M taurocholic acid (Sigma-Aldrich) for 1 h to release sporozoites. Sporozoites (2.5 × 10^5^) were added to each well of a 96-well plate. Chicken NK-lysin (Genscript, Piscataway, NJ) at concentrations of 1.0, 10, and 100 μg/mL were used as positive controls. Three different maltol doses, low (0.1 μg/mL), medium (1.0 μg/mL), and high (10 μg/mL), were used to treat freshly prepared live sporozoites and incubated at 41°C for 3 h. Fluorescent dye (AO/PI staining solution, Nexcelom Bioscience LLC, Lawrence, MA) was added to each mixture in a 1:1 ratio, and live sporozoites were counted using a cell counting chamber (Nexcelom Bioscience LLC). All experiments were replicated more than three times independently.

#### QMC Culture

QMCs (2 × 10^5^/mL) were seeded in 24-well plates, as previously described (20) in Medium 199 (Hyclone) containing 10% FBS and 1% penicillin/streptomycin until 70% confluence was achieved. Media in 12-wells were replaced by Medium 199 containing 0.5% FBS with 1% penicillin/streptomycin to induce cell differentiation, and in the remaining 12-wells of the same plate, media were replaced by a basic Medium 199 containing 10% FBS to maintain cell proliferation. Maltol at concentrations of 0.0, 0.1, 1.0, and 10.0 μg/mL was added to each well in the 24-well plates. After incubation at 41°C in a humidified atmosphere with 5% CO_2_ for 18 h, all cells were harvested in lysis buffer with 2-mercaptoethanol. RNA was extracted using QIAcube for performing qRT-PCR analysis. All experiments were replicated more than three times independently.

#### Primary Chicken Embryonic Muscle Cell (PMC) Culture

Eggs for the embryo were obtained from Moyer's hatchery (Quakertown, PA). The PMC culture was modified based on the method described by Hassan et al. (25). Briefly, eggs were incubated at 41°C and 80% humidity. The pectoralis major region of the embryos was extracted at 13 days; it was minced and digested with 0.05% trypsin-EDTA (Sigma-Aldrich) at 37°C for 20 min. The primary cells were washed 2–3 times with Hanks balanced salt solution (Sigma-Aldrich) and seeded (2 × 10^5^/mL) in 24-well plates. Primary muscle cells were incubated in DMEM (Hyclone) containing 10% FBS and 1% penicillin/streptomycin until 70% confluence was achieved. Media in 12-wells were replaced by DMEM containing 2% FBS with 1% penicillin/streptomycin to induce cell differentiation, and in the remaining 12-wells of the same plate, media were replaced by basic DMEM containing 10% FBS to maintain cell proliferation. Maltol at concentrations of 0.0, 0.1, 1.0, and 10.0 μg/mL was added to each well in the 24-well plates. After incubation at 41°C in a humidified atmosphere with 5% CO_2_ for 18 h, all cells were harvested in lysis buffer with 2-mercaptoethanol. RNA was extracted using QIAcube for qRT-PCR analysis. All experiments were replicated more than three times.

#### Analysis of Cytokines, Tight Junction (TJ) Proteins, and Markers of Muscle Cell Growth by qRT-PCR

The levels of pro-inflammatory cytokines (IL-1β, IL-6, and IL-8) were determined in IEC and CMC using extracted RNA samples. For analysis of TJ proteins (occludin, ZO-1, and MUC2), qRT-PCR was conducted using RNA samples extracted from IEC without LPS stimulation. Proliferation and differentiation markers of muscle cells, Pax7 and MyoG, were measured using samples obtained from QMC and PMC. Total RNA was extracted using QIAcube. RNA was eluted in 30 μL RNase-free water, and the concentration and yield were determined using the NanoDrop ND-1000 spectrometer (Nano-Drop Technology, Wilmington, DE, USA). A 1-μg aliquot of total RNA was reverse-transcribed to cDNA using the QuantiTect Reverse Transcription Kit (Qiagen), and all cDNA samples were diluted 1:10 with RNase-free water for subsequent analysis. qRT-PCR was performed using the Agilent Mx3000 P QPCR System (Agilent Technologies, Santa Clara, CA) and the Brilliant SYBR Green qRT-PCR Master Mix (Stratagene, La Jolla, CA). Oligonucleotide primer sequences and accession numbers used for chicken cytokines are listed in Table 1. Standard curves were generated using log10 diluted standard samples of RNA to calculate the amplification efficiency, and the levels of individual transcripts were normalized to those of β-actin using the Q-gene software program (26).

**Table 1:** Oligonucleotide primer sequences for qRT-PCR.

**Type**	**Target gene**	**Primer sequence (5^**′**^-3^**′**^)**	**PCR product size (Kb)**
Reference	GAPDH	F-GGTGGTGCTAAGCGTGTTAT	264
		R-ACCTCTGCCATCTCTCCACA	
Pro-inflammatory	IL-1β	F-TGGGCATCAAGGGCTACA	244
		R-TCGGGTTGGTTGGTGATG	
	IL-6	F-CAAGGTGACGGAGGAGGAC	254
		R-TGGCGAGGAGGGATTTCT	
	IL-8	F-GGCTTGCTAGGGGAAATGA	200
		R-AGCTGACTCTGACTAGGAAACTGT	
	IL-17F	F-TGAAGACTGCCTGAACCA	117
		R-AGAGACCGATTCCTGATGT	
	TNFSF15	F-CCTGAGTATTCCAGCAACGCA	292
		R-ATCCACCAGCTTGATGTCACTAAC	
Th1	IFN-γ	F-AGCTGACGGTGGACCTATTATT	259
		R-GGCTTTGCGCTGGATTC	
	IL-10	F-CGGGAGCTGAGGGTGAA	272
		R-GTGAAGAAGCGGTGACAGC	
TJ proteins	Claudin-1	F- CCTGATCACCCTCTTGGGAG	145
		R- GCTGCACTCACTCATTGGCT	
	JAM2	F-AGCCTCAAATGGGATTGGATT	59
		R-CATCAACTTGCATTCGCTTCA	
	Occludin	F-GAGCCCAGACTACCAAAGCAA	68
		R-GCTTGATGTGGAAGAGCTTGTTG	
	ZO-1	F-CCGCAGTCGTTCACGATCT	63
		R-GGAGAATGTCTGGAATGGTCTGA	
Muscle cell	MyoG	F-TGACCCTGTGCCCTGAAAGC	178
		R-TCGTTCACCTTCTTCAGCCTCC	
	Pax7	F-AAGGCCAAGCACAGCATAGA	108
		R-GCGCTGCTTCCTCTTCAAAG	

*Th, T helper cells; TJ, tight junction*.

### Experiment 2: *In vivo* Study

**Figure 1:**
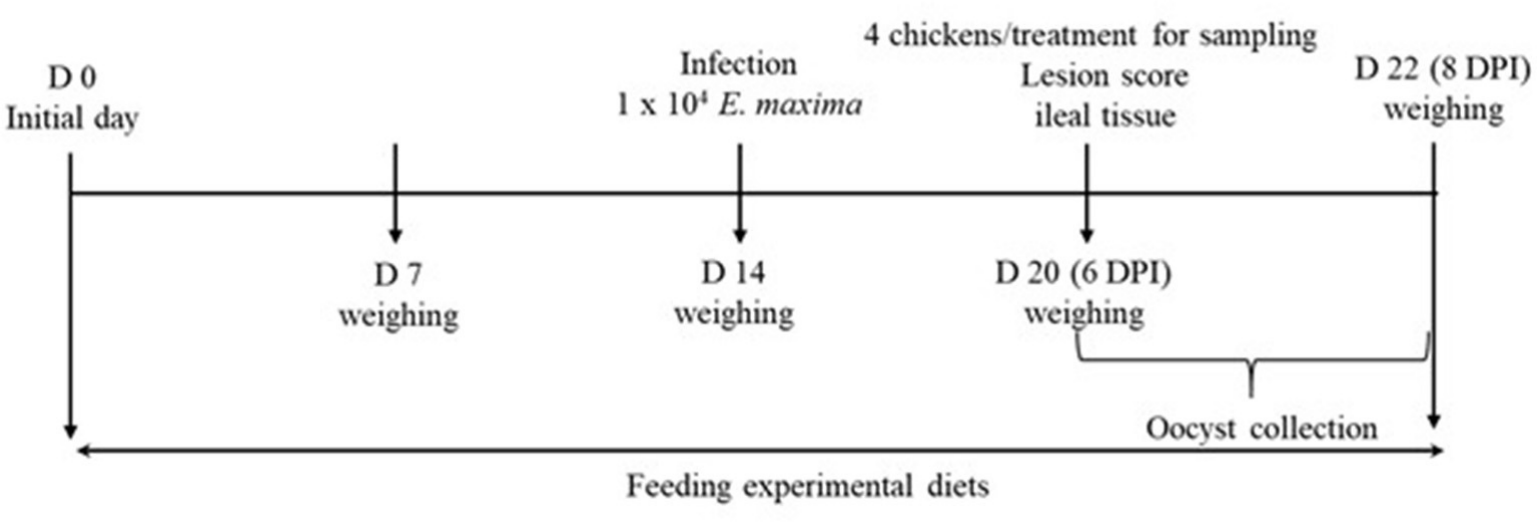
Schematic outline of the experimental design in experiment 2.

All animal experiments were approved by the Beltsville Agricultural Research Center Institutional Animal Care and Use Committee (# 19–018). Figure 1 depicts the schematic outline of the experimental design used for this study.

#### Chickens and Experimental Design

A total of 80 newly hatched (Ross 708) male broiler chickens at 0 days of age were purchased from Longenecker's hatchery (Elizabethtown, PA). The day after the chickens arrived at the Beltsville ARS facility, they were weighed to perform adjustments to obtain the same body weight (BW) per treatment and allocated to four dietary treatments in a randomized complete block design. The dietary treatments included provision of a corn- and soybean meal-based (basal) diet without infection (CON, Table 2), basal diet with *E. maxima* infection (NC), maltol (Sigma-Aldrich) at 10.0 mg/kg feed (HI), and maltol at 1.0 mg/kg feed (LO) Each treatment group was allocated into two cages with ten chickens per cage (0.65 × 0.75 m^2^). The chickens were provided with *ad libitum* access to water and feed throughout the experimental period. A schematic outline of the experimental design is shown in Figure 1.

**Table 2:** Ingredient composition of basal diet (as feed? basis, %, unless otherwise indicated).

**Ingredients (%)**	**Basal diet**
Corn	55.78
Soybean meal	37.03
Soybean oil	2.97
Dicalcium phosphate	1.80
Calcium carbonate	1.51
Salt	0.38
Poultry Vit Mix^a^	0.22
Poultry Mineral Mix^b^	0.15
DL-Methionine	0.10
Choline-chloride, 60%	0.06
Total	100
**Calculated values (%)**	
CP, %	24.00
Ca, %	1.20
AP, %	0.51
Total Lys, %	1.40
Total Met, %	0.49
Total Cys + Met, %	0.80
ME, Mcal/kg	3.5

a *Vitamin mixture provided the following nutrients per kg of diet: vitamin A, 2,000 IU; vitamin D3, 22 IU; vitamin E, 16 mg; vitamin K, 0.1 mg; vitamin B1, 3.4 mg; vitamin B2, 1.8 mg; vitamin B6, 6.4 mg; vitamin B12, 0.013 mg; biotin, 0.17 mg; pantothenic acid, 8.7 mg; folic acid, 0.8 mg; niacin, 23.8 mg*.

b *Mineral mixture provided the following nutrients per kg of diet: Fe, 400 mg; Zn, 220 mg; Mn, 180 mg; Co, 1.3 mg; Cu, 21 mg; Se, 0.2 mg. CP, crude protein; AP, available phosphorus*.

#### Determination of BW

Chickens were weighed on days 1, 7, 14, 20, and 22 for the computation of BW and average daily gain (ADG). Dead chickens were removed and weighed daily to perform adjustments for the growth data.

#### Oral Infection With *E. maxima*

All chickens, except for those in the CON group, were orally infected with *E. maxima* (1.0 × 10^4^ oocysts/bird; Beltsville strain 41A) on day 14 as previously described (27). The purity of the infected *E. maxima* was confirmed by conducting a DNA genotyping test (28).

#### Collection of Intestinal Samples

Two chickens from each cage with a medium BW were euthanized by cervical dislocation on day 20, and their intestines were removed for further analysis. From each intestine, a small section (2 cm) of the jejunum without contents was collected aseptically and stored in RNAlater® (Invitrogen, Carlsbad, CA) at −20°C until subsequent analysis.

#### Gut Lesion Scoring

Using the 15-cm long mid jejunum sample, gut lesion scoring was performed on day 20. Lesions were scored on a scale from 0 (none) to 4 (high) by four independent observers in a blinded manner, as per methods described previously (29).

#### Fecal Oocyst Shedding

From days 20–22 (6–8 days post-infection: dpi), fecal samples were collected and the number of sporulated oocysts was determined as previously described (30) using the McMaster chamber according to the following formula:

Total oocysts/chicken = [oocyst count × dilution factor × (fecal sample volume/counting chamber volume)]/number of chickens per cage.

#### Isolation of RNA and Reverse Transcription

Total RNA was isolated from jejunum samples that were stored in RNAlater® according to the manufacturer's recommendations. Approximately 50 mg of the jejunal tissue was homogenized in 1 mL of TRIzol (Invitrogen) using a hand-held homogenizer (TissueRuptor; Qiagen). Chloroform was added to the homogenized sample. The samples were centrifuged at 12,000 × *g* for 15 min at 4°C for phase separation. RNA present in the colorless upper aqueous phase was then precipitated using 100% isopropanol (Sigma-Aldrich). The RNA pellet was washed with 75% ethanol (Sigma-Aldrich), air-dried, and re-suspended in RNase-free water (Invitrogen). The quantity of RNA was assessed using the NanoDrop (ND-1000) spectrophotometer (NanoDrop Products, Wilmington, DE) according to the absorbance at 260 nm. RNA purity was evaluated based on the OD260/OD280 ratio. Total RNA (1 μg) was then reverse-transcribed to cDNA using the QuantiTect® reverse transcription kit (Qiagen). Briefly, the RNA sample was incubated with genomic DNA wipeout buffer at 42°C for 2 min to remove any genomic DNA contamination. Reverse transcription (RT) of the genomic DNA-depleted sample was performed by the addition of the Quantiscript Reverse Transcriptase, Quantiscript RT buffer, and RT primer mix (Qiagen). The reaction was performed in a thermal cycler (Mastercycler® EP Gradient S; Eppendorf, Hauppauge, NY). The cycling conditions were 42°C for 30 min, followed by reverse transcriptase inactivation at 95°C for 3 min. The cDNA samples were divided into aliquots and stored at – 20°C.

#### Gene Expression Analysis by qRT-PCR

The oligonucleotide primer sequences used for qRT-PCR are listed in Table 1. The expression of various cytokines and intestinal TJ proteins was evaluated in the jejunum, including IL-1β, IL-6, IL-10, IL-17F, IFN-γ, TNFSF15, JAM2, occludin, ZO-1, and claudin-1. Glyceraldehyde-3-phosphate dehydrogenase was used as the reference gene. Amplification and detection were performed using the Stratagene Mx3000P qPCR system (Agilent Technologies Inc.) and RT^2^ SYBR Green qPCR master mix (Qiagen). Each sample was analyzed in triplicate, and non-specific primer amplification was assessed via the inclusion of no-template controls. Standard curves were generated using log10 diluted standards of RNA, and the levels of individual transcripts were normalized to those of Glyceraldehyde-3-phosphate dehydrogenase in the Q-gene program (26).

#### Statistical Analysis

*In vitro* data for each response were analyzed using the Proc GLM in SAS (SAS Inc., Cary, NC). *In vivo* data were analyzed using a mixed model (PROC MIXED) in SAS. Each chicken was considered an experimental unit. The results are shown as least squares mean values and pooled standard error of the mean. Probability values <0.05 were considered significantly different. In cases where the overall effect was significant, the mean values were compared in a pairwise manner (PDIFF option).

## Results

### Experiment 1: *In vitro* Studies

#### Effect of Maltol on the Gene Expression of Biomarkers Associated With Inflammation and Gut Integrity in Chicken Epithelial Cells

**Figure 2:**
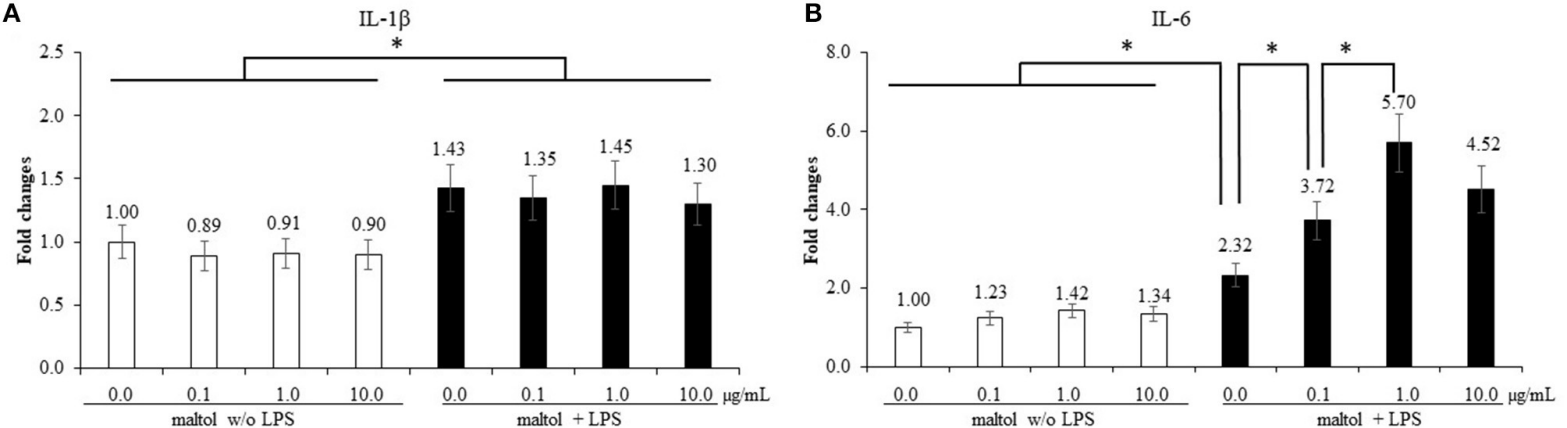
Secretion of pro-inflammatory cytokines in chicken epithelial cells (IEC) by LPS and maltol. Each bar represents the mean ± SEM (*n* = 3). Transcript levels of the cytokines were measured using quantitative RT-PCR and normalized to GAPDH transcript levels. Significant results are marked as * (*P* < 0.05). LPS, lipopolysaccharide.

**Figure 3:**
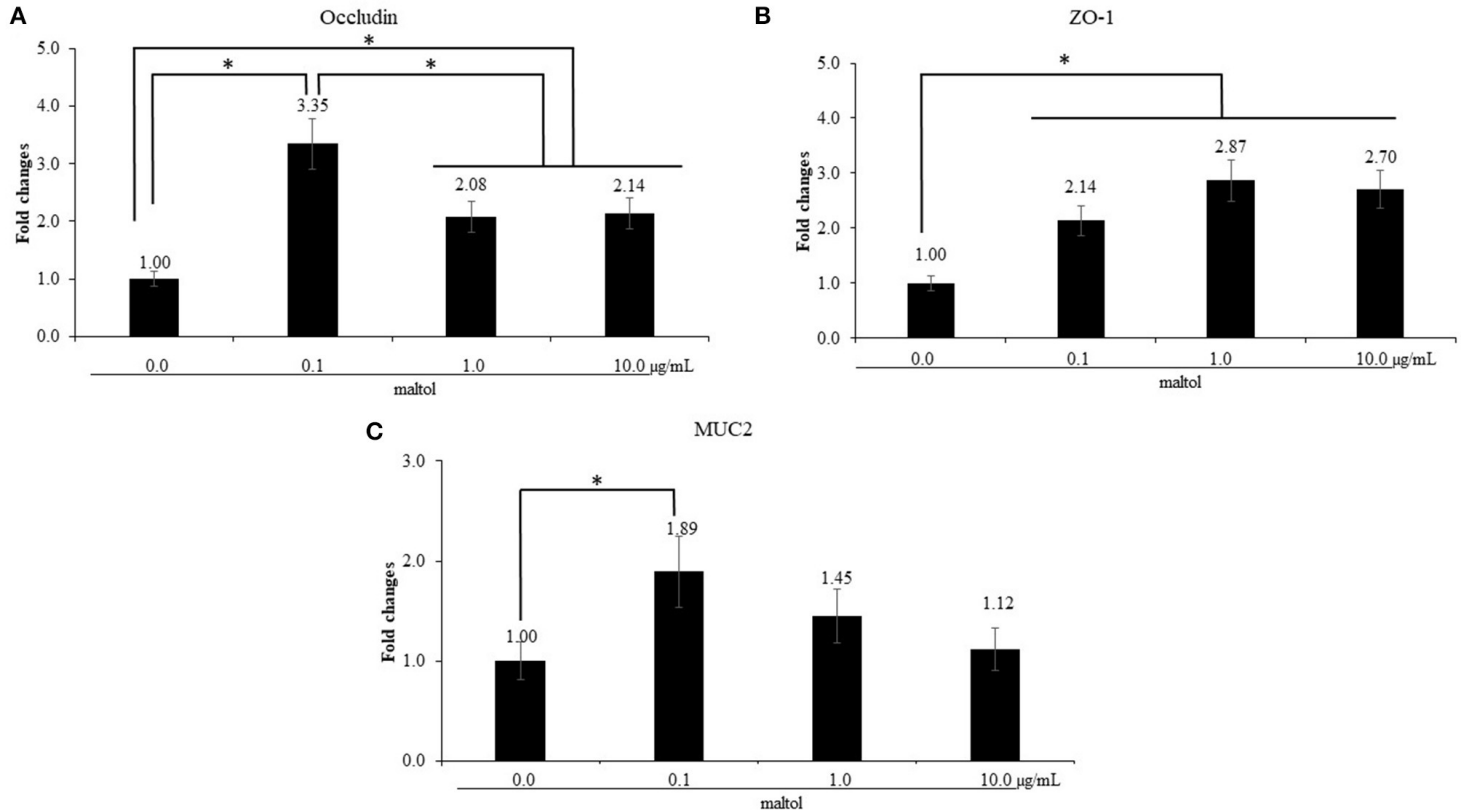
Alteration of tight junction proteins and mucin in chicken epithelial cells (IEC) by maltol. Each bar represents the mean ± SEM (*n* = 3). Transcript levels of the tight junction proteins were measured using quantitative RT-PCR and normalized to GAPDH transcript levels. Significant results are marked as * (*P* < 0.05).

LPS stimulation of IEC increased (*P* = 0.041) IL-1β levels (0.9- to 1.4-fold) compared to those in the control without LPS treatment, whereas maltol alone did not change the IL-1β levels (Figure 2A). For IL-6, LPS stimulation increased (*P* = 0.038) IL-6 levels (1.3- to 4.1-fold) independent of maltol treatment compared to those of the groups without LPS stimulation (Figure 2B). With LPS treatment, maltol increased (*P* < 0.002) IL-6 levels in a dose-dependent manner from 0.1 (2.32- to 3.7-fold) to 1.0 μg/mL (2.3- to 5.7-fold) compared to those of the LPS control (maltol at 0.0 μg/mL). Maltol increased occludin (Figure 3A, 1.0- to 3.4-fold) and MUC2 (Figure 3C, 1.0- to 1.9-fold) gene expression (*P* < 0.001) at a dose of 1.0 μg/mL. Maltol also induced higher (*P* < 0.001) ZO-1 levels (1.0- to 2.6-fold) from 0.1 to 10.0 μg/mL in IEC than those in the control without maltol (Figure 3B).

#### Effect of Maltol on Pro-inflammatory Responses of CMC

**Figure 4:**
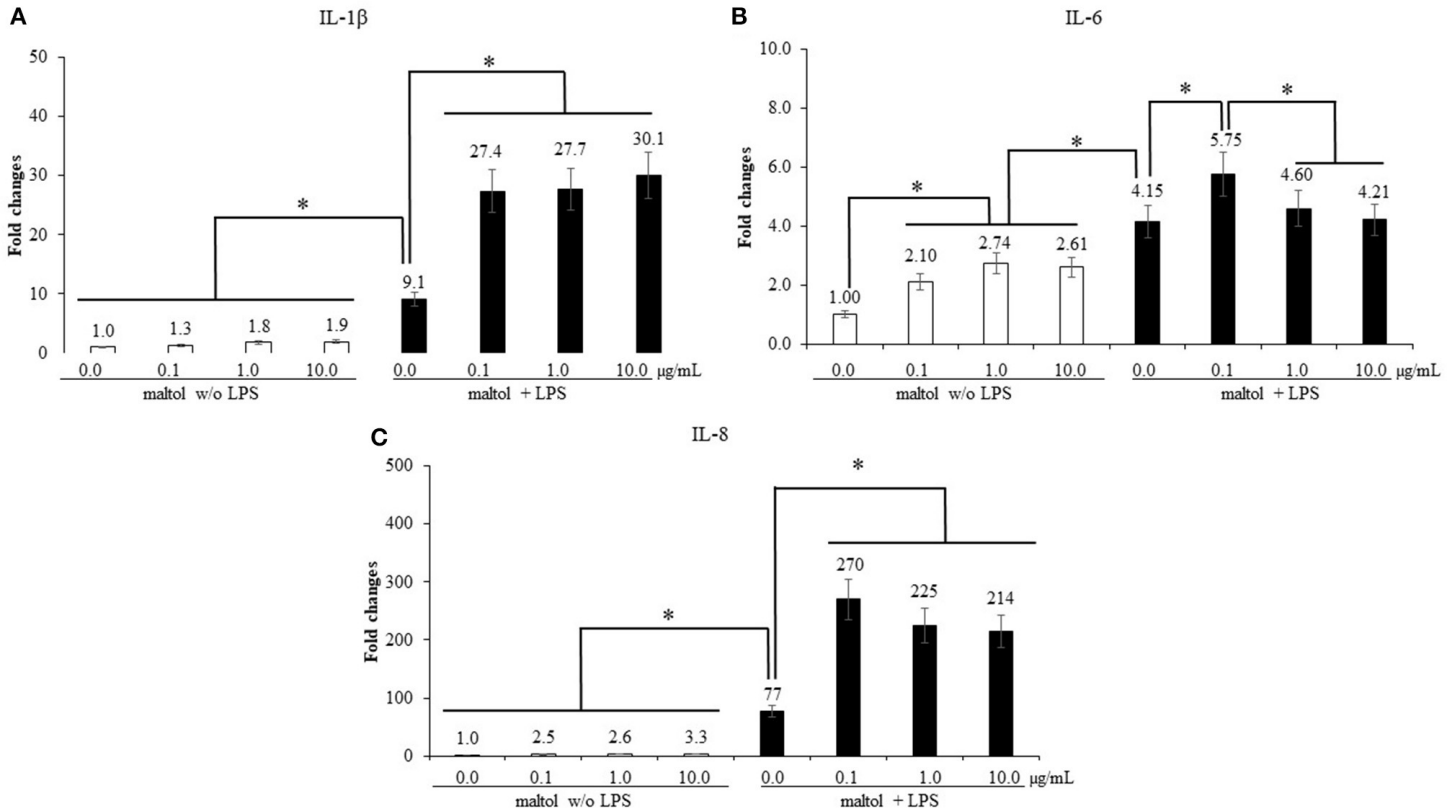
Secretion of pro-inflammatory cytokines in chicken macrophages (CMC) by LPS and maltol. Each bar represents the mean ± SEM (*n* = 3). Transcript levels of the cytokines were measured using quantitative RT-PCR and normalized to GAPDH transcript levels. Significant results are marked as * (*P* < 0.05). LPS, lipopolysaccharide.

LPS stimulation increased (*P* < 0.001) IL-1β levels (1.5- to 9.1-fold) in CMC compared to those in the groups without LPS treatment, and maltol treatment further increased (*P* < 0.001) IL-1β levels from 9.1- to 28.4-fold (Figure 4A). In groups without LPS stimulation, maltol increased IL-6 levels from 0.1 to 10.0 μg/mL (*P* < 0.042) (1.0- to 2.5-fold) compared to those in the control (Figure 4B). LPS stimulation increased (*P* = 0.038) IL-6 levels (2.1- to 4.5-fold) compared to those of groups without LPS stimulation. Among the LPS groups, maltol at a dose of 0.1 μg/mL increased (*P* = 0.028) IL-6 levels (4.2- to 5.8-fold) compared to those in the groups with LPS administration without maltol (Figure 4B). Furthermore, LPS stimulation increased (*P* < 0.001) IL-8 levels (2.4- to 77-fold) in CMC compared to those in non-LPS groups (Figure 4C). Among the LPS groups, maltol at a dose of 0.1 μg/mL increased (*P* = 0.048) IL-8 levels (77- to 270-fold) compared to those in the LPS control without maltol treatment.

#### Anticoccidial Activity of Maltol Against Sporozoites of *E. maxima*

**Figure 5:**
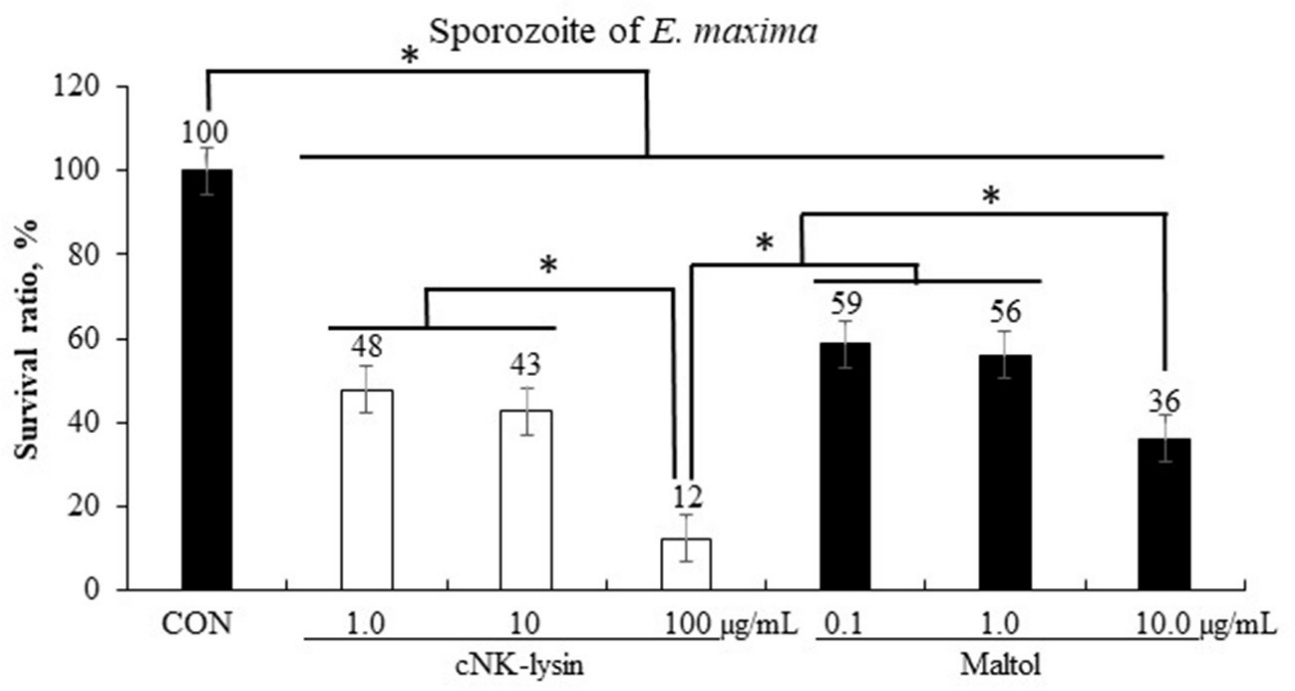
Anticoccidial effect of maltol on sporozoites of *E. maxima* in experiment 1. Each bar represents the mean ± SEM (*n* = 3). Significant results are marked as * (*P* < 0.05). CON, 2.5 × 10^5^ sporozoites/mL.

Chicken NK-lysin which was used as a positive control decreased (*P* < 0.001) the survival rate of sporozoites of *E. maxima* in a dose-dependent manner by 12–48% compared to that in the CON group (Figure 5). Maltol showed a dose-dependent decrease of sporozoites of *E. maxima* by 36–59% compared to that in the CON group.

#### Effect of Maltol on the Proliferation and Differentiation of QMCs and PMCs

**Figure 6:**
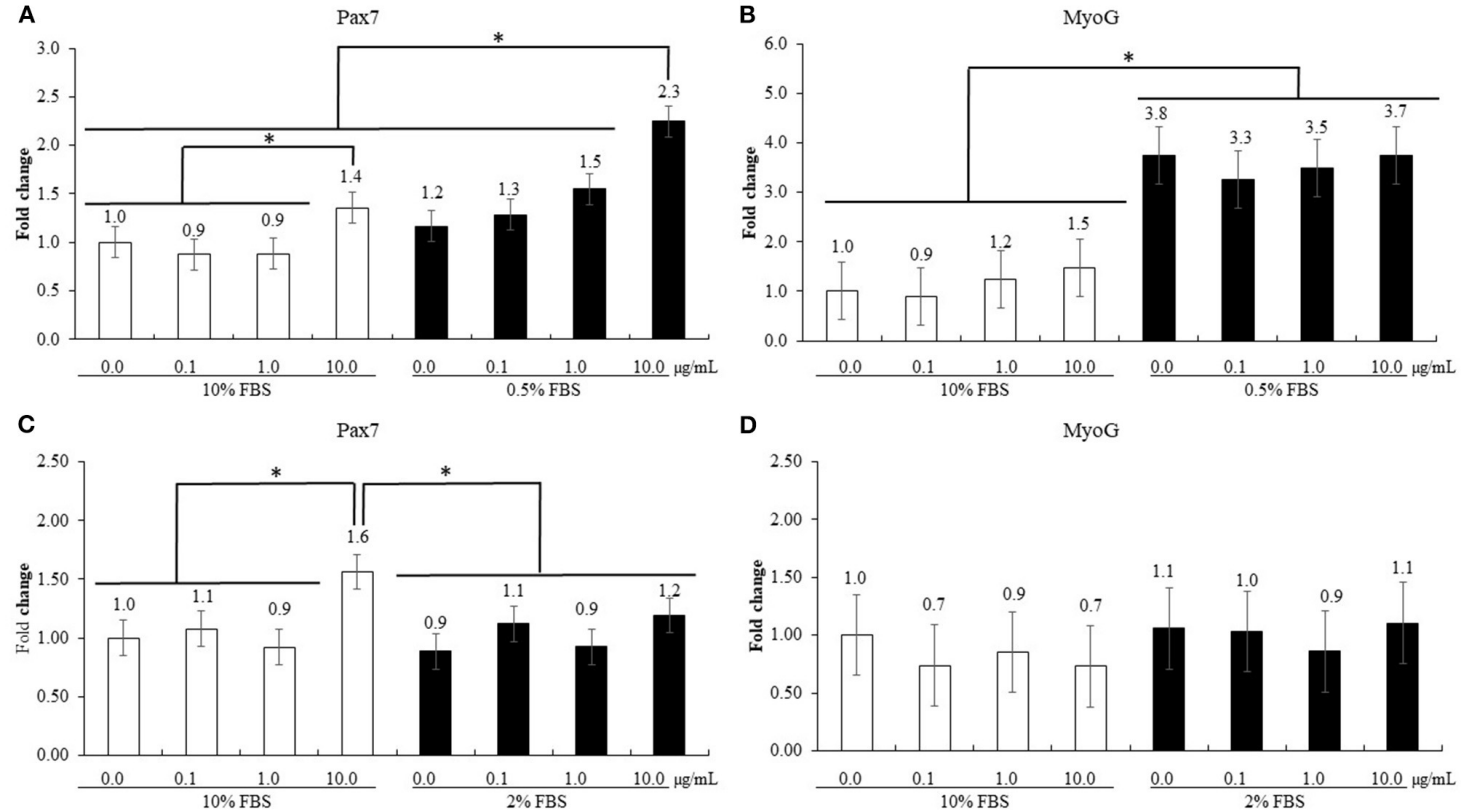
Proliferation and differentiation of quail muscle cells [QMC, (**A,B**)] and primary chicken embryonic muscle cells [PMC, **(C,D)**] by FBS concentration and maltol. Each bar represents the mean ± SEM (*n* = 3). Transcript levels of the cytokines were measured using quantitative RT-PCR and normalized to GAPDH transcript levels. Significant results are marked as follows: * (*P* < 0.05).

The treatment using 0.5% FBS without maltol did not change (*P* > 0.05, 1.0–1.2) Pax7 levels of QMCs compared to those observed after treatment using 10% FBS (Figure 6). However, maltol at a dose of 10.0 μg/mL increased (*P* < 0.05) Pax7 levels regardless of the FBS concentration compared to those in the corresponding control groups (1.0–1.4 in 10% FBS and 1.2–2.3 in 0.5% FBS). In contrast, in the treatment groups using 0.5% FBS, maltol increased (*P* < 0.05) the MyoG levels (1.2–3.6) of QMCs regardless of the dose of maltol used compared to those observed after treatment using 10% FBS. In PMC, maltol at a dose of 10.0 μg/mL increased Pax7 levels (1.0–1.6) in the group subjected to a treatment using 10% FBS (*P* < 0.05) compared to those in the other groups. However, MyoG levels of PMCs did not change (*P* > 0.05) with respect to the maltol dose or FBS concentration used.

### Experiment 2: *In vivo* Studies

#### Growth Performance of Chickens

The initial BW did not show significant differences among the experimental groups (*P* = 0.998; Table 3). In the absence of challenge infection, treatment with dietary maltol did not significantly change (*P* > 0.05) the BW of chickens until day 14 regardless of the dose of maltol used compared to that of the control group, even though the HI and LO groups increased the BW of chickens numerically compared to those in the CON and NC groups. *E. maxima* infection decreased (*P* < 0.001) the BW of chickens at 6 dpi (860–743 g) and 8 dpi (1,017–763 g) compared to that of the CON group. The HI group increased the BW of chickens at 6 dpi (*P* = 0.040, 743–794 g) and 8 dpi (*P* < 0.001, 763–934 g) compared to that of the NC group. The LO group also increased (*P* = 0.015) the BW of chickens (763–842 g) at 8 dpi compared to that of the NC group. Before infection, the ADG of the chickens fed with diets supplemented with maltol did not differ (*P* > 0.05) from that of the CON group, although the ADGs of chickens fed with diets supplemented with maltol increased numerically regardless of the dose administered. *E. maxima*-infected chickens (NC group) presented with lower ADG at 0 to 6 dpi (*P* < 0.001, 66.5–47.7 g) compared to that of the uninfected CON group. The HI group increased the ADG of the infected chickens at 0–6 dpi (*P* = 0.011, 47.7–53.7 g). During the entire infection period (0–8 dpi), the HI (*P* < 0.001, 38.2–57.8 g) and LO (*P* = 0.002, 38.2–46.9 g) groups increased the ADG of chickens compared to that of the NC group, which was decreased by *E. maxima* infection.

**Table 3:** Body weight and average daily gain chickens fed a diet supplemented with maltol.

**Treatment**	**CON**	**NC**	**HI**	**LO**	**SEM**	***P*-value**
**BW, g**						
Initial	36.6	36.6	36.6	36.6	0.7	0.998
D 7	157	159	161	162	2.7	0.651
D 14 (0 dpi)	461	457	472	473	8.8	0.509
D 20 (6 dpi)	860^a^	743^c^	794^b^	724^c^	17	< 0.001
D 22 (8 dpi)	1,017^a^	763^d^	934^b^	842^c^	22	<0.001
**ADG, g**						
D 0 to 7	20.0	20.4	21.0	20.8	0.3	0.169
D 7 to 14	43.5	41.9	44.4	44.6	1.0	0.214
D 0 to 14^1^	32.7	32.2	33.3	33.6	0.6	0.434
D 14 to 20	66.5^a^	47.7^c^	53.7^b^	41.9^d^	1.6	<0.001
D 14 to 22^2^	69.4^a^	38.2^d^	57.8^b^	46.9^c^	1.9	<0.001

*CON, basal diet; NC, basal diet for E. maxima-infected chickens; HI, maltol at 10.0 mg/kg feed; LO, maltol at 1.0 mg/kg feed; SEM, standard error of the mean (n = 20/treatment until day 20 and 16/treatment at day 22)*.

1 *Before infection*.

2 *After infection*.

*ADG, average daily gain; BW, body weight; D, day; dpi, days post-infection; all chickens except CON were infected by oral gavage at day 14 with 1.0 × 10^4^ oocysts/chicken of E. maxima*.

a−d *Means in the same row with different superscripts differ (P < 0.05) and the difference was re-evaluated by PDIFF option in SAS when P-value between treatments was < 0.05*.

#### Intestinal Lesion Scores and Fecal Oocyst Shedding

**Figure 7:**
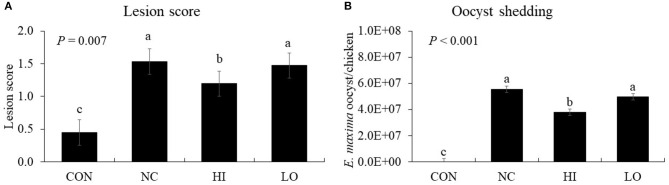
Lesion score and oocyst shedding of chickens fed diet supplemented with maltol during infection with *E. maxima*. CON, basal diet; NC, basal diet for infected chickens; HI, maltol at 10.0 mg/kg feed; LO, maltol at 1.0 mg/kg feed; all chickens except CON were infected by oral gavage at day 14 with 1.0 × 10^4^ oocysts/chicken of *E. maxima*. ^a−c^Bars with no common letter differ significantly (*P* < 0.05). Each bar represents the mean ± SEM (*n* = 4). The lesion score was collected from distal jejunal tissue at day 20 (6 days post-infection: dpi) and fecal sample was collected from 6 to 8 dpi to calculate the oocyst shedding.

*E. maxima* infection increased (*P* < 0.001) the gut lesion scores (0.4–1.5) of the jejunum compared to that of the control group (Figure 7A). The HI group decreased (*P* = 0.024) the lesion score of the jejunum (1.5–1.2) compared to that of the NC group. In the NC group, infection increased (*P* < 0.001) the oocyst numbers (0–5.6 × 10^7^ oocysts/chicken) compared to that of the CON group; however, the HI group decreased (*P* = 0.002) the fecal oocyst number (5.6 × 10^7^-3.8 × 10^7^ oocysts/chicken) compared to that of the NC group (Figure 7B).

#### Pro-inflammatory Cytokines

**Figure 8:**
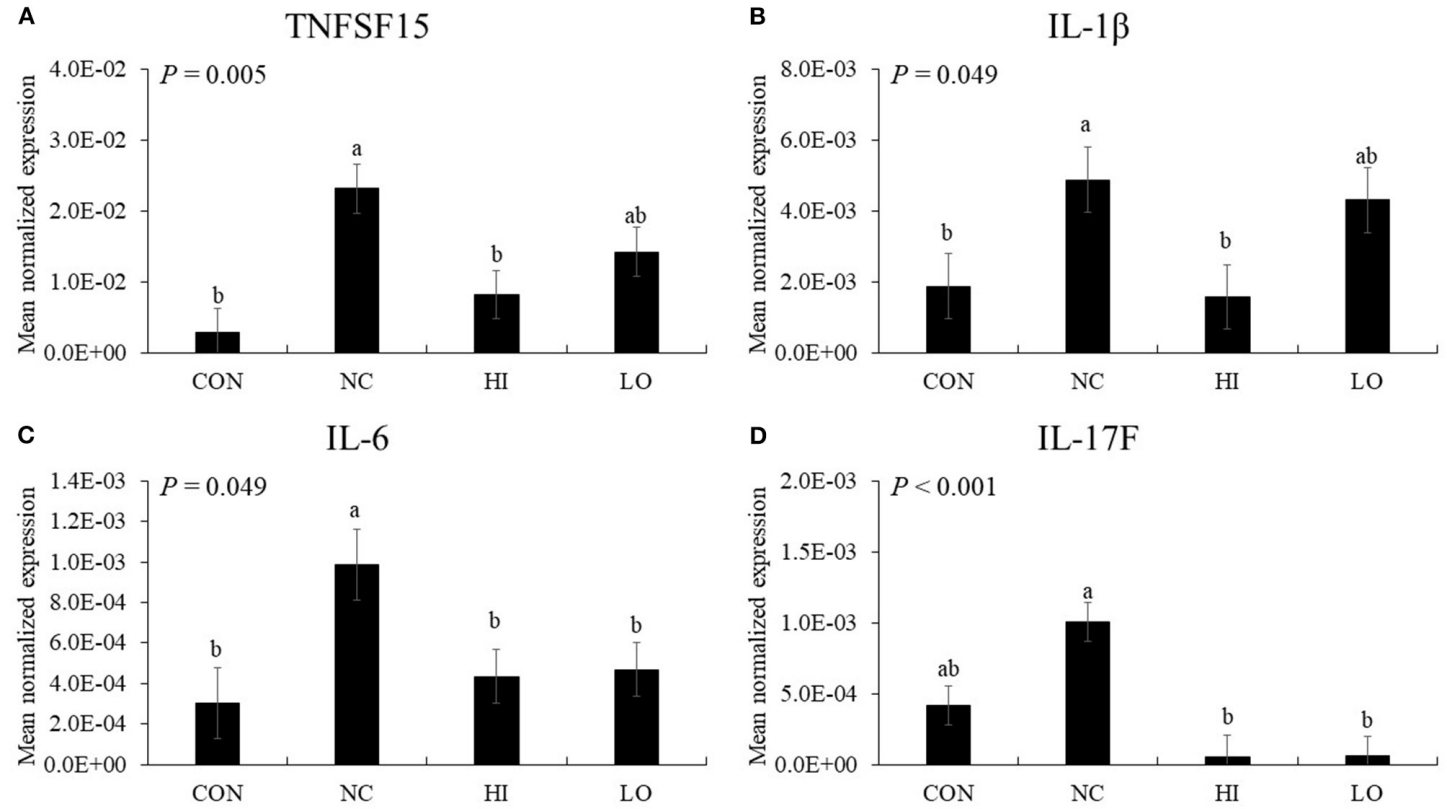
Transcripts of proinflammatory cytokines in jejunum of chickens fed diet supplemented with maltol during infection with *E. maxima* in experiment 2. CON, basal diet; NC, basal diet for infected chickens; HI, maltol at 10.0 mg/kg feed; LO, maltol at 1.0 mg/kg feed; all chickens except CON were infected by oral gavage at day 14 with 1.0 × 10^4^ oocysts/chicken of *E. maxima*. ^a,b^Bars with no common letter differ significantly (*P* < 0.05). Each bar represents the mean ± SEM (*n* = 4). The data were collected at day 20 (6 days post-infection). Transcript levels of the cytokines were measured using quantitative RT-PCR and normalized to GAPDH transcript levels.

In the absence of maltol, infection (NC group) with *E. maxima* increased (*P* < 0.05) the gene expression of TNFSF15 (3.4 × 10^−3^-2.3 × 10^−2^), IL-1β (1.9 × 10^−3^-4.9 × 10^−3^), and IL-6 (3.0 × 10^−3^-9.9 × 10^−3^) in the distal jejunum compared to that of the CON group (Figure 8). The HI group decreased (*P* < 0.05) the expression of TNFSF15 (2.3 × 10^−2^-8.2 × 10^−3^), IL-1β (4.9 × 10^−3^-1.6 × 10^−3^), IL-6 (9.9 × 10^−3^-4.4 × 10^−3^), and IL-17F (1.0 × 10^−3^-5.0 × 10^−5^) compared to that of the NC group. The LO group also decreased (*P* < 0.05) the gene expression of IL-6 (9.9 × 10^−3^-4.7 × 10^−3^) and IL-17F (1.0 × 10^−3^-6.0 × 10^−5^) compared to that of the NC group.

#### Th1 Cytokines

**Figure 9:**
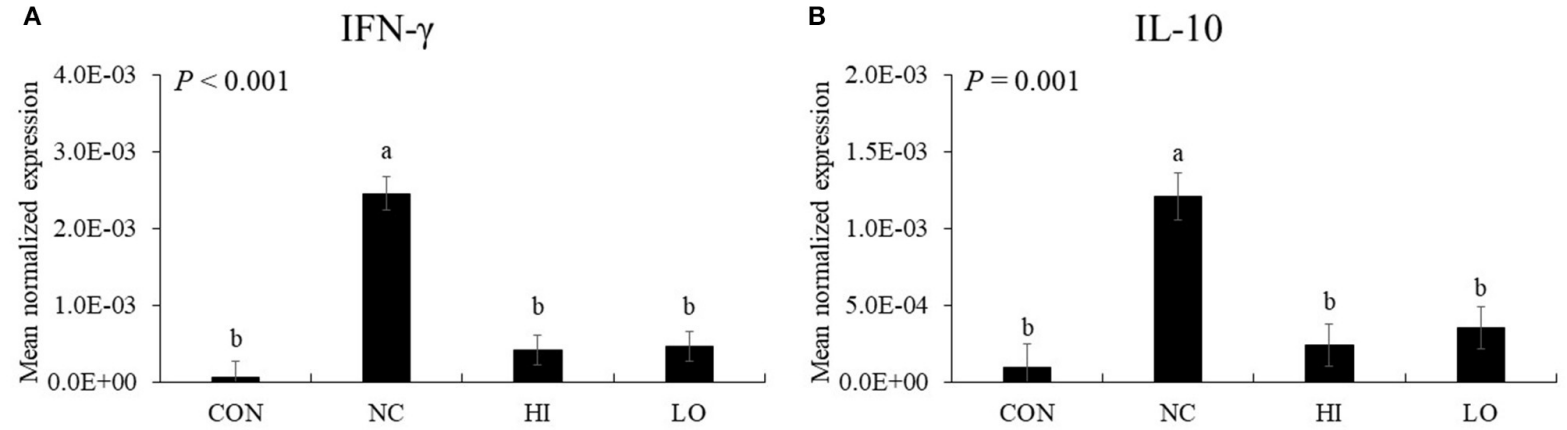
Transcripts of Th1 cytokines in jejunum of chickens fed diet supplemented with maltol during infection with *E. maxima* in experiment 2. CON, basal diet; NC, basal diet for infected chickens; HI, maltol at 10.0 mg/kg feed; LO, maltol at 1.0 mg/kg feed; all chickens except CON were infected by oral gavage at day 14 with 1.0 × 10^4^ oocysts/chicken of *E. maxima*. ^a,b^Bars with no common letter differ significantly (*P* < 0.05). Each bar represents the mean ± SEM (*n* = 4). The data were collected at day 20 (6 days post-infection). Transcript levels of the cytokines were measured using quantitative RT-PCR and normalized to GAPDH transcript levels.

In the absence of maltol, challenge infection (NC group) with *E. maxima* increased (*P* < 0.05) the gene expression of IFN-γ (5.1 × 10^−5^-2.5 × 10^−3^) and IL-10 (9.1 × 10^−5^-1.2 × 10^−3^) in the distal jejunum compared to that of the CON group (Figure 9). However, the HI and LO groups decreased (*P* < 0.05) the gene expression of IFN-γ (HI: 2.5 × 10^−3^-4.1 × 10^−4^, and LO: 2.5 × 10^−3^-4.6 × 10^−4^) and IL-10 (HI: 1.2 × 10^−3^-2.4 × 10^−4^ and LO: 1.2 × 10^−3^-3.5 × 10^−4^) compared to their respective NC controls.

#### TJ Proteins

**Figure 10:**
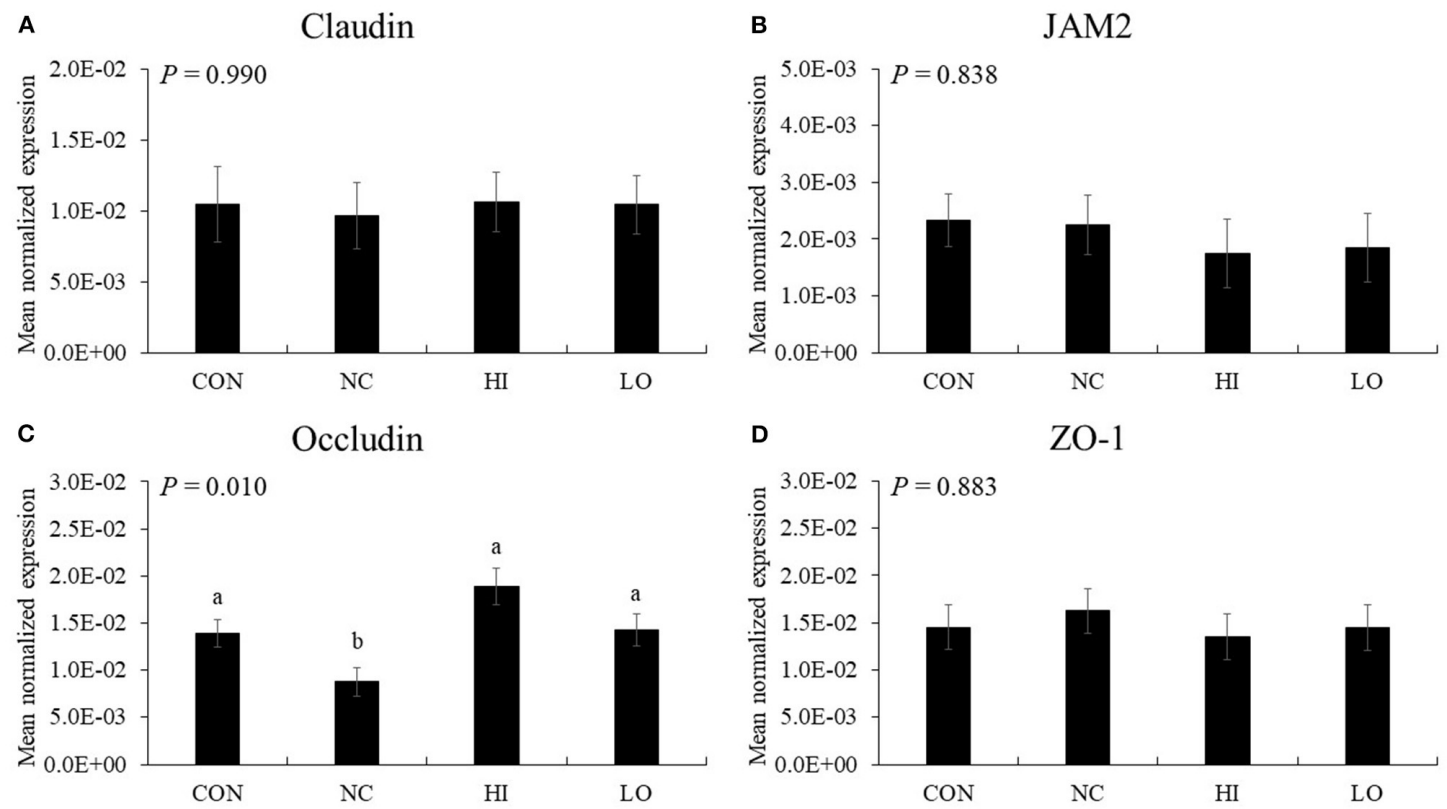
Transcripts of tight junction protein in jejunum of chickens fed diet supplemented with maltol during infection with *E. maxima* in experiment 2. CON, basal diet; NC, basal diet for infected chickens; HI, maltol at 10.0 mg/kg feed; LO, maltol at 1.0 mg/kg feed; all chickens except CON were infected by oral gavage at day 14 with 1.0 × 10^4^ oocysts/chicken of *E. maxima*. ^a,b^Bars with no common letter differ significantly (*P* < 0.05). Each bar represents the mean ± SEM (*n* = 4). The data were collected at day 20 (6 days post-infection). Transcript levels of the tight junction proteins were measured using quantitative RT-PCR and normalized to GAPDH transcript levels.

In the absence of maltol, challenge infection (NC group) with *E. maxima* decreased (*P* = 0.033) the gene expression of occludin (1.4 × 10^−2^-8.8 × 10^−3^) in the distal jejunum compared to that in the CON group (Figure 10). However, the HI (8.8 × 10^−3^-1.9 × 10^−2^) and LO (8.8 × 10^−3^-1.4 × 10^−2^) groups increased (*P* < 0.031) occludin gene expression compared to that of the NC group. Other TJ proteins such as claudin-1, JAM2, and ZO-1 did not demonstrate any significant changes in gene expression (*P* > 0.05) following maltol treatment.

## Discussion

Analysis of metabolites produced by gut microbiota-mediated metabolism of indigestible feed ingredients has provided evidence of profound effects of small molecular weight metabolites on the signal transduction of the host and as regulators of host physiological responses (18, 31, 32). Recently, several metabolites have been named as postbiotics, which are defined as types of feed additives that are generally produced by beneficial gut microbes and exert a positive effect on host health (18). For example, short-chain fatty acids, bacteriocins, functional peptides, and proteins are known as postbiotics (33, 34); therefore, gut metabolites are considered as postbiotics. In our previous study (19), we performed a global metabolomic analysis of gut metabolites associated with growth and immunity enhancement following dietary exposure of young chickens with direct-fed microbes, *B. subtilis* 1781 and 747, which promote growth and immunity. Of the 361 metabolites that were altered in chickens treated with *B. subtilis* 1781 and 747, 131 metabolites were increased and 230 metabolites were decreased compared to the non-supplemented control group. Maltol was identified as one of the markedly increased metabolites with potentially beneficial effects on gut health and chicken growth.

In experiment 1, the effects of maltol on the gene expression of pro-inflammatory cytokines in IEC and CMC, TJ proteins in IEC, and anticoccidial ability against sporozoite of *E. maxima*, were evaluated *in vitro*. LPS was used to induce inflammatory responses in IEC and CMC, and its treatment elevated levels of all pro-inflammatory cytokines tested. The magnitude of cytokine gene expression was greater in CMC than that in IEC. In IEC, IL-1β and IL-6 levels were increased by 1.4 and 2.3 times, respectively, compared to those in each control without LPS treatment, whereas in CMC, IL-1β, IL-6, and IL-8 levels were increased by 9.1, 4.2, and 7.7 times, respectively, compared to their respective controls. Therefore, CMC was more responsive to the LPS treatment than to the IEC treatment, and the findings were supported by a previous study which showed that macrophages were more sensitive to LPS-induced immune responses than epithelial cells (35). LPS treatment of IEC and CEC in the present study increased expression levels of all cytokines analyzed, and maltol, at doses from 0.1 μg/mL (1.6-fold higher than the LPS control) to 1.0 μg/mL (2.5-fold higher than the LPS control), induced IL-6 levels of IEC in a dose-dependent manner. However, maltol at a dose of 10 μg/mL maintained the same level of IL-6 expression as that observed at doses of 0.1 and 1.0 μg/mL. For CMC, regardless of the dose administered, maltol induced higher IL-1β and IL-8 levels compared to those of the LPS control without maltol addition. IL-6 showed the most remarkable change at the lowest maltol concentration (0.1 μg/mL) among the LPS-treated groups. Other maltol concentrations (1.0 and 10.0 μg/mL) did not affect the LPS-induced IL-6 levels.

Our *in vitro* studies showed that maltol played a role as an immune modulator during inflammatory responses. Maltol also influences the anti-oxidant activity (22) by scavenging free radicals generated during oxidative stress (20). Oxidative stress incurred during an inflammatory response may trigger the overproduction of reactive oxygen species, which can form a vicious circle resulting in tissue damage in intestinal epithelia and liver tissues (36). Maltol treatment has shown effective protection of nerve cells against oxidative damage caused by reactive oxygen species (33), and maltol-derived organometallic complexes have also been shown to demonstrate potential anti-tumor activity (21). Pro-inflammatory cytokines are involved in mucosal inflammation, disruption of the physiological epithelial TJ structure and composition, and can increase paracellular permeability (37). However, the molecular mechanisms that drive TJ reorganization in such inflammatory states are not well-understood. Only maltol in the absence of LPS upregulated the gene expression of occludin, ZO-1, and MUC2 levels in IEC by more than 2-fold, regardless of the dose administered compared to the control. Based on our observations, maltol may exert a beneficial effect on maintenance of gut integrity and improve the gene expression of TJ proteins. Further studies are warranted to clarify the role of maltol and TJ proteins in IEC.

Maltol also reduced the survival ratio of sporozoites of *E. maxima*, although the underlying reason for this needs further investigation. A previous study showed that 1-(N-acetyl-6-aminohexyl)-3-hydroxy-2-methylpyridib-one, synthesized from maltol, inhibited the growth of *Plasmodium falciparum* by interfering with the uptake of exogenous iron or by depleting the intracellular labile iron pool (38). Since both *Eimeria* and *Plasmodia* belong to the Apicomplexa phylum, maltol may use a similar mechanism to inhibit the growth of these protozoan parasites. The observed effects of maltol on muscle cells *in vitro* warrant further study. In the present study, we used different FBS concentrations [0.5% FBS in QMC (39) and 2% FBS in PMC (40)] to induce myogenic differentiation and proliferation. During myoblast proliferation, maltol administration at a dose of 10 μg/mL enhanced Pax7 levels in QMCs and PMCs. However, regardless of the dose administered, maltol treatment did not affect the MyoG levels of QMCs and PMCs during myogenic differentiation. Maltol at a dose of 10 μg/mL also increased Pax7 levels in QMCs during myogenic differentiation. Choi et al. (41) reported that high levels of Pax7 could impair myogenesis formation. According to our observation, QMCs and PMCs present with different cell growth properties because PMCs are undifferentiated primary cells obtained from chicken embryos, whereas QMCs are established muscle cells obtained from quails.

To further study the role of maltol in chickens, we conducted an *in vivo* feeding trial in young broiler chickens infected with *E. maxima*. Specifically, the effects of dietary maltol at high and low doses on growth performance, intestinal immune responses, and epithelial barrier integrity of chickens following challenge infection with *E. maxima* were examined. Regardless of the dose administered, dietary maltol supplementation in young chickens did not affect the BW and ADG before *E. maxima* infection. Therefore, dietary maltol supplementation in broiler chicken may not influence the growth of chickens by itself. However, in the present study, dietary maltol supplementation up to 10 μg/kg feed did not adversely affect the growth performance of young broiler chickens. After infection with *E. maxima*, dietary maltol supplementation showed various changes in the physiological responses of broiler chickens. Following *E. maxima* infection, the growth performance (BW and ADG) of the broiler chickens were decreased compared to that of the CON group regardless of the dietary maltol supplemented. However, at 6 dpi, the BW of chickens were improved when chickens were provided with dietary HI maltol levels of 10.0 μg/kg, and the effects could be observed until the end of the experiment. When chickens were provided with LO maltol levels of 1.0 μg/kg feed, BW showed improvement only at 8 dpi. The effect of dietary maltol supplementation on BW was also investigated to determine changes in the ADG of chickens, with the HI group showing improvements in the ADG between 0 and 8 dpi (the entire infection duration). These results indicate the beneficial effects of dietary maltol on the BW and ADG of chickens following *E. maxima* infection. The beneficial effect of maltol on muscle growth is an important finding which suggests a potential use of maltol as an alternative to antibiotics. Therefore, further studies are needed to investigate the mechanisms of growth promotion by maltol since the focus of current study was to show the effect of dietary maltol on gut function including local immunity in broiler chickens.

The results of our *in vitro* and *in vivo* experiments supported our hypothesis that dietary maltol supplementation could improve the growth of broiler chickens infected with *E. maxima* and indicated that maltol was a good candidate as a postbiotic antibiotic alternative. To further investigate the effect of dietary maltol on the growth performance of chickens, we determined jejunal lesion score, gene expression of jejunal cytokines and TJ proteins, and fecal oocyst shedding in broiler chickens infected with *E. maxima*. Dietary maltol supplementation decreased the jejunal lesion score and fecal oocyst production, indicating a potential direct role of dietary maltol on *Eimeria* fecundity or host cell invasion at the brush border of the intestine. This result was supported by our *in vitro* results showing the direct action of maltol on the survival of sporozoites of *E. maxima* in the present study. Another explanation for the decrease in gut lesions and fecal oocyst shedding could be provided by the effects of maltol treatment on cytokine and TJ protein expression in the gut. Expression levels of the cytokines, which are mostly involved in the elicitation of pro-inflammatory and Th1 responses, increased after challenge infection with *E. maxima*. However, following *E. maxima* infection, dietary maltol supplementation suppressed the gene expression levels of IL-6, IL-17F, IFN-γ, and IL-10 compared to those of the NC group. These results suggest a role played by dietary maltol in induction of host immune responses and oocyst reduction during *E. maxima* infection.

Klasing (42) has reported that a cytokine storm induces metabolic changes, including increased protein degradation in skeletal muscle, thereby diverting nutrients from the muscle and other tissues to render their availability for the increased demands of leukocytes and production of protective proteins. Consequently, these responses decrease growth performance and directly influence the success of poultry production. In practice, under equalized feed intake, a vigorous acute phase immune response in chickens has been estimated to account for approximately 10% of the nutrient use (42). Jiang et al. (43) reported that LPS-challenged chickens (dose of 1 mg LPS per kg of BW at 14, 16, 18, and 20 days of age) showed a 22% decrease in BW gain during challenge; 59% of the loss was attributed to a decreased feed intake, and the remaining 41% was attributed to the presence of immune response-related factors (44). Park et al. (3) suggested that cytokines that reduced pro-inflammatory and cellular immune responses could decrease the turnover of feed nutrients utilized for immune protein production, which might lead to exertion of growth-promoting effects on chickens. This concept is also supported by studies and reviews related to nutrition and immunity in poultry (43–45). The intestinal epithelium, composed of a single layer of columnar epithelial cells that are tightly bound by intercellular junctional complexes, serves as a physical barrier against invading pathogens and intraluminal toxins (46, 47). Intestinal junctional complexes maintain the integrity of the epithelial barrier by regulating paracellular permeability and are composed of TJs, gap junctions, adherens junctions, and desmosomes (48). TJs include four integral transmembrane proteins (occludin, claudin-1, JAM, and tricellulin) that interact with cytosolic scaffold proteins, which bind the actin cytoskeleton (46, 49). Jejunal TJ proteins levels, except those for occludin, were not affected by *E. maxima* infection or dietary maltol supplementation in the present study. However, occludin levels were decreased by *E. maxima* infection, and the reduction was recovered by dietary maltol supplementation in a dose-dependent manner. Our results were supported by findings reported by Van Itallie et al. (50), who stated that occludin was required for cytokine-induced TJ remodeling, however, dietary maltol did not affect other TJ proteins unlike our *in vitro* results. Thus, further studies on the effects of maltol on TJ proteins will enhance our understanding of maltol-gut junction protein interaction.

In conclusion, maltol, one of the metabolites whose expression was increased in the gut of young broiler chickens fed with a diet supplemented with *B. subtilis* 1781 and 747, that promotes host immunity and growth, affected the maintenance of gut integrity and the functions of immunity *in vitro* and exerted beneficial effects by reducing intestinal damage, parasite fecundity, and inflammatory responses in chickens following *E. maxima* challenge infection. Therefore, maltol is a good candidate as an antibiotic alternative postbiotic to improve the growth and immunity of chickens presenting with enteric diseases.

## Data Availability Statement

The original contributions presented in the study are included in the article/supplementary material, further inquiries can be directed to the corresponding author/s.

## Ethics Statement

The animal study was reviewed and approved by the Beltsville Agricultural Research Center Small Animal Care Committee (Animal Protocol No. 19-018).

## Author Contributions

IP and HL designed the research and draft the manuscript and edit. IP and SW conducted the research. IP, DG, HN, SW, and KL analyzed data. IP, DG, HN, SW, KL, NZ, AS, TR, and HL had responsibility for content. All authors contributed to the article and approved the submitted version.

## Conflict of Interest

NZ, AS, and TR were employed by the Arm & Hammer Animal and Food Production. The remaining authors declare that the research was conducted in the absence of any commercial or financial relationships that could be construed as a potential conflict of interest.
